# Contemporary Scurvy in Vulnerable Populations in High-Income Countries: A Systematic Review and Multilevel Meta-Analysis

**DOI:** 10.3390/nu18142213

**Published:** 2026-07-08

**Authors:** Nina Cadeau-Comte, Erika Meléndez-Oliva, Juan Nicolás Cuenca-Zaldívar, Eleuterio A. Sánchez-Romero

**Affiliations:** 1 Interdisciplinary Research Group on Musculoskeletal Disorders, 28014 Madrid, Spain; nina.c@etik.com; 2 Université Toulouse–Jean Jaurès, 5 Allées Antonio Machado, 31058 Toulouse Cedex 9, France; 3 Grupo de Investigación en Dietética Aplicada, Nutrición y Composición Corporal (DANuC), Department of Optics, Pharmacology and Anatomy, University of Alicante, 03690 Alicante, Spain; 4 Physiotherapy and Orofacial Pain Working Group, Sociedad Española de Disfunción Craneomandibular y Dolor Orofacial (SEDCYDO), 28009 Madrid, Spain; 5 Research Group in Nursing and Health Care, Puerta de Hierro Health Research Institute-Segovia de Arana (IDIPHISA), 28222 Majadahonda, Spain; 6 Physical Therapy Unit, Primary Health Care Center El Abajón, 28231 Las Rozas de Madrid, Spain; 7 Department of Rehabilitation Sciences, Florida Gulf Coast University, Fort Myers, FL 33965, USA

**Keywords:** scurvy, vitamin C deficiency, pediatrics, systematic review, meta-analysis, high-income countries, musculoskeletal, neurodevelopmental disorders, case report

## Abstract

**Background**: Scurvy, a condition caused by vitamin C deficiency, has traditionally been considered eradicated in high-income countries (HICs; World Bank classification). However, emerging evidence suggests its re-emergence within identifiable vulnerability clusters, challenging the conventional assumptions about nutritional adequacy in affluent settings. This study aimed to systematically characterize the contemporary clinical and epidemiological profile of scurvy in HICs and quantify its multisystem burden. **Methods**: A systematic review and meta-analysis were conducted following the Preferred Reporting Items for Systematic Reviews and Meta-Analyses (PRISMA) 2020 guidelines and registered in the International Prospective Register of Systematic Reviews (PROSPERO; CRD420251013454). PubMed, Embase, Web of Science, and Scopus were searched (2012–2025). Case reports, case series, and observational studies reporting clinically or biochemically confirmed scurvy in HICs were included in this review. A multilevel random-effects meta-analysis was performed using restricted maximum likelihood estimation, incorporating robust variance estimation to account for within-study dependence and hierarchical data structures. Multisystem involvement was modeled as a count outcome, and system-specific prevalence was estimated using logit-transformed proportions. **Results**: Twenty-five studies, including 147 patients, were analyzed. Patients diagnosed with scurvy frequently presented with involvement of multiple organ systems, with a pooled mean of 2.29 affected organ systems per patient (95% CI: 1.62–2.96; *I*^2^ = 66.8%), highlighting clinically relevant heterogeneity in presentation. Musculoskeletal involvement was the most frequent clinical domain (OR 1.82; 95% CI: 0.98–3.38), whereas neurological manifestations were significantly less common (OR 0.42; 95% CI: 0.28–0.62; *p* < 0.001). Age was the only significant moderator, showing a modest inverse association with musculoskeletal involvement (OR 0.97; *p* = 0.035). Across studies, cases predominantly clustered in pediatric patients with neurodevelopmental disorders, restrictive dietary patterns, medically complex conditions, and psychosocial vulnerability. **Conclusions**: Scurvy persists in high-income countries as a clinically under-recognized condition occurring predominantly in vulnerable populations with restrictive dietary patterns, neurodevelopmental disorders, and psychosocial vulnerability. The available evidence suggests that contemporary scurvy is frequently associated with multisystem manifestations; however, the contribution of concomitant micronutrient deficiencies cannot be excluded. These findings highlight the need for comprehensive nutritional assessment and targeted nutritional screening in high-risk populations to prevent diagnostic delays and avoidable morbidity.

## 1. Introduction

Scurvy, historically known as the “plague of the sea,” is a clinical condition resulting from severe vitamin C (ascorbic acid) deficiency. First described in ancient civilizations, it became particularly prominent during the Age of Exploration, when prolonged maritime voyages led to characteristic manifestations such as gingival bleeding, petechial rash, impaired wound healing, and musculoskeletal pain [1,2]. The seminal work of James Lind in 1747, which demonstrated the preventive role of citrus fruits, was a cornerstone in the development of nutritional medicine and the identification of dietary deficiency-related diseases [3].

Although vitamin C thresholds vary in the literature, important distinctions exist between subclinical insufficiency, biochemical deficiency, and clinically overt scurvy. Plasma vitamin C concentrations below approximately 11 µmol/L are generally considered compatible with severe biochemical deficiency associated with the clinical manifestations of scurvy, whereas higher thresholds have been proposed to define hypovitaminosis C or subclinical depletion states. In the present review, the <11 µmol/L threshold was adopted when biochemical confirmation was reported, as this cutoff was the most consistently associated with clinically manifest scurvy in the contemporary clinical literature.

With the development of modern food systems and improved nutritional availability, vitamin C deficiency has traditionally been considered rare in high-income countries. The current dietary reference intakes (DRIs) for vitamin C vary slightly across high-income regions. For example, the recommended intake for adult men is approximately 90 mg/day in the United States and 110 mg/day in several European countries, including Spain, levels that are generally achievable through the routine consumption of fruits and vegetables [4,5]. However, recent clinical reports and hospital-based case series suggest a re-emergence of scurvy in high-income countries (HICs; World Bank classification), particularly among individuals with restrictive dietary patterns, neurodevelopmental disorders, or significant psychosocial vulnerability [6,7,8,9,10].

According to the World Bank, high-income countries are those with a gross national income per capita exceeding $12,696 [11]. Despite the presence of advanced healthcare systems, structured food supply chains, and public health nutrition programs, disparities in access to adequate nutrition persist in these populations. Recent population-based nutritional surveillance data from the United States have demonstrated that vitamin C deficiency remains prevalent in vulnerable subgroups, particularly among individuals with low dietary intake, smoking habits, obesity, and socioeconomic disadvantage, reinforcing concerns regarding persistent micronutrient inequities even in high-income settings [12]. These persistent deficiency patterns are clinically relevant because vitamin C depletion may manifest through heterogeneous multisystem presentations, including musculoskeletal, mucocutaneous, hematological, and neurological manifestations, frequently contributing to delayed or missed diagnoses. Certain groups, including individuals with autism spectrum disorders, children with selective eating behaviors, elderly individuals living alone, patients with psychiatric conditions, and those experiencing food insecurity, may be disproportionately affected by micronutrient deficiencies, including scurvy [13,14,15,16,17].

Understanding the contemporary epidemiology of scurvy in HICs is important for several reasons [18,19,20]. First, this may reflect persistent health inequities within affluent societies. Second, it highlights emerging risk profiles, such as neurodevelopmental disorders, eating disorders, and extreme dietary restrictions, which differ from the historically recognized causes of the disease [6,13,14,15,16,17,18,19,20]. Third, it underscores the need to maintain a clinical suspicion of nutritional deficiencies in both pediatric and adult populations, particularly in psychiatric, oncological, and socially vulnerable contexts [13,14,15,16,17,18,19,20]. Finally, improved characterization of these patterns may inform targeted public health strategies aimed at preventing avoidable vitamin C deficiency [18,19,20].

Although scurvy classically presents with gingival bleeding, follicular hyperkeratosis, corkscrew hairs, and impaired wound healing, recent studies have emphasized a broader and often atypical clinical spectrum [21]. Reported manifestations include limping, refusal to walk, unexplained fatigue, irritability, bone pain, and clinical features that may mimic rheumatologic, infectious, or malignant conditions [10,17,18,19,22,23]. Consequently, delayed diagnosis remains common, particularly when clinicians do not initially consider scurvy in the differential diagnosis [24,25,26], leading to delays that may extend for several weeks to months. These diagnostic challenges are especially pronounced in patients with neurodevelopmental disorders, psychiatric comorbidities, and marked social vulnerability [13,14,15,16,17,24,25,26].

The current body of evidence on contemporary scurvy in HICs is largely composed of case reports and small case series, with relatively few observational studies including multiple patients [2,6,7,8,9,10,15,16,17,18,19,20]. This fragmented and hierarchically structured evidence base, characterized by the clustering of multiple cases within individual studies, poses significant challenges for conventional evidence synthesis and limits the ability to derive robust quantitative estimates of disease burden [2,6,7,8,9,10,15,16,17,18,19,20]. In this context, multilevel meta-analytic approaches incorporating robust variance estimation have been proposed as a methodological strategy to account for within-study dependence and between-study heterogeneity, enabling a more comprehensive assessment of multisystem involvement across the life course [2,6,7,8,9,10,15,16,17,18,19,20]. This emerging pattern of nutritional vulnerability within resource-rich environments has not yet been quantitatively characterized in the literature.

Unlike previous predominantly narrative descriptions of isolated contemporary scurvy cases, the present study aimed to quantitatively synthesize the multisystem clinical expression, contextual vulnerability profiles, and diagnostic implications of scurvy in high-income countries using a multilevel meta-analytic framework.

Against this background, we conducted a systematic review and meta-analysis of contemporary scurvy in high-income countries between 2012 and 2025. The objectives of this study were to (1) comprehensively characterize the available evidence base, (2) describe the main clinical and contextual patterns across affected individuals, (3) summarize diagnostic approaches and treatment strategies, and (4) quantify the multisystem burden of scurvy while exploring potential moderators of system-specific involvement throughout the life course.

## 2. Materials and Methods

### 2.1. Protocol and Registration

This systematic review and meta-analysis was conducted in accordance with the Preferred Reporting Items for Systematic Reviews and Meta-Analyses (PRISMA) 2020 statement [27]. The protocol was prospectively registered in the International Prospective Register of Systematic Reviews (PROSPERO) under registration number CRD420251013454.

### 2.2. Eligibility Criteria

Eligible studies were primary reports describing clinically and/or biochemically diagnosed scurvy in populations residing in countries classified as high-income by the World Bank [11] at the time of publication. Case reports, case series, retrospective cohort studies, and other observational designs were eligible if they provided original patient-level data or extractable subgroup-level data on clinical presentation, contextual factors, diagnosis, treatment, or outcomes. To capture contemporary evidence, the search was restricted to studies published between January 2012 and March 2025.

Studies were excluded if they met any of the following criteria: (1) historical reports published before 2010; (2) studies conducted exclusively in low- or middle-income countries; (3) review articles, editorials, conference abstracts without extractable primary data, letters, or narrative summaries; (4) animal or in vitro studies; or (5) reports restricted to isolated dental manifestations without sufficient systemic information or with incomplete data precluding data extraction.

Low- and middle-income countries (LMICs) were defined according to the World Bank income classification framework as economies with a gross national income per capita below the threshold established for high-income countries during the study period.

### 2.3. Information Sources and Search Strategy

A comprehensive literature search was conducted using PubMed/MEDLINE, Embase, Web of Science, and Scopus databases. The searches covered the period from 1 January 2012 to 31 March 2025 and combined controlled vocabulary and free-text terms related to scurvy, vitamin C deficiency, ascorbic acid, pediatric and adult populations, malnutrition, and high-income settings. The search strategy is provided directly below in the main text.

The PubMed strategy was as follows:

(“Scurvy” [MeSH Terms] AND “Vitamin C Deficiency” [MeSH Terms] OR “ascorbic acid deficiency” [All Fields] OR “hypovitaminosis C” [All Fields]) AND (“Child” [MeSH Terms] OR “Infant” [MeSH Terms] OR “Adolescent” [MeSH Terms] OR “Pediatrics” [MeSH Terms] OR “Aged” [MeSH Terms] OR “Elderly” [All Fields]) AND (“Malnutrition” [MeSH Terms] OR “Food Insecurity” [MeSH Terms] OR “Feeding and Eating Disorders” [MeSH Terms] OR “Selective Eating” [All Fields]) AND (“Systemic Diseases” [MeSH Terms] OR “Systemic Symptoms” [All Fields] OR “Public Health” [MeSH Terms] OR “Epidemiology” [MeSH Terms] OR “Health Disparities” [MeSH Terms]).

The gray literature was explored through Google Scholar, and the reference lists of the included studies and relevant reviews were hand-searched to identify further eligible articles. All retrieved citations were imported into a reference manager, and duplicates were removed using a combination of automated and manual checks before screening.

### 2.4. Study Selection

All retrieved records were imported into a reference manager and screened for duplicates. Two reviewers (N.C.C. and E.M.O.) independently screened the titles and abstracts against predefined eligibility criteria. Full-text articles considered potentially eligible were independently assessed by the same reviewers. Any disagreements were resolved by discussion and, when necessary, by consultation with a third reviewer (E.A.S.-R.) until a consensus was reached.

### 2.5. Data Collection Process

Data extraction was performed independently by two reviewers using a standardized, piloted extraction form. For each included study, the following variables were extracted when available: (1) study characteristics (authors, year of publication, country, study design, and healthcare setting); (2) patient demographics (age, sex, and socioeconomic context); (3) contextual and risk factors (dietary patterns, neurodevelopmental or psychiatric disorders, oncological treatment, comorbidities, and social vulnerability); (4) diagnostic approach (clinical criteria, biochemical confirmation, and imaging findings); (5) clinical manifestations across six predefined organ systems (musculoskeletal, cutaneous, gingival/mucosal, hematological, systemic, and neurological); (6) treatment details (route and dose of vitamin C supplementation and adjunctive interventions); and (7) outcomes (time to improvement, complications, and relapse).

When studies reported subgroup-level or aggregate data rather than fully disaggregated individual patient data, separate extraction lines were created and coded for incorporation into the multilevel analysis framework. Discrepancies in data extraction were resolved by consensus, and the corresponding authors were contacted when clarification of key variables was necessary.

### 2.6. Risk-of-Bias Assessment

The methodological quality of the included studies was assessed using the Joanna Briggs Institute (JBI) Critical Appraisal Checklists for case reports and case series [28] and the ROBINS-I tool for non-randomized observational studies [29].

Given the expected predominance of descriptive and retrospective designs, these tools were selected to provide design-specific evaluations of methodological rigor rather than to infer the overall certainty of the evidence.

Two reviewers (E.M.O. and N.C.C.) independently assessed the risk of bias in each study. Discrepancies were resolved through discussion and, when necessary, adjudication by a third reviewer (E.A.S.-R.).

The results of the quality assessment are graphically summarized to facilitate comparisons across study designs.

### 2.7. Quantitative Synthesis and Statistical Analysis

All statistical analyses were conducted by J.N.C.-Z. using R software (version 4.1.3; R Foundation for Statistical Computing, Vienna, Austria), following the published methodological guidelines for meta-analyses [30]. Given the heterogeneous structure of the evidence base, including single-patient case reports, case series, and retrospective observational cohorts, a multilevel random-effects meta-analytic framework was used to account for the clustering of multiple observations within studies and the dependence between effect sizes derived from the same source.

The primary quantitative outcome was the multisystem burden of scurvy, which was operationalized as the number of affected organ systems in each patient. This count-based outcome was modeled within a multilevel random-effects framework using restricted maximum likelihood (REML) estimation. Between-study heterogeneity was assessed using the τ^2^ statistic, Cochran’s Q test, and *I*^2^ index. Heterogeneity was interpreted as unimportant (<30%), moderate (30–50%), substantial (50–75%), or considerable (>75%).

A prediction interval was calculated to indicate the expected range of the true effects in comparable future populations. Inference was based on small-sample-adjusted procedures, including Hartung–Knapp-type corrections where appropriate [31].

Given the nesting of the data, categorical subgroup analyses were replaced by univariable and multivariable multilevel meta-regressions using candidate clinical and methodological moderators to comprehensively explore sources of total heterogeneity (I^2^). For each of the six predefined clinical systems (musculoskeletal, cutaneous, gingival/mucosal, hematological, systemic, and neurological), multivariable meta-regression models were fitted using logit-transformed proportions as the dependent variables. Candidate moderators were selected a priori on clinical and methodological grounds and included age, restrictive diet, neurodevelopmental disorder, biochemical confirmation of vitamin C deficiency (≤11 µmol/L), use of diagnostic imaging (X-ray or MRI), time from symptom onset to diagnosis (weeks), data source type (individual case-based vs. cohort aggregate), and hospital size. Exponentiated estimates were reported as odds ratios (ORs) or incidence rate ratios (IRRs) for the corresponding model parameterization.

Standard meta-analyses assume that each included study provides a single, independent data point. However, our dataset features a hierarchical (multilevel) structure, where multiple individual patient cases and their respective clinical signs are nested within the same primary study. Because patients described within the same article often share identical clinical settings, diagnostic criteria, and geographic backgrounds, their data exhibit internal correlation (i.e., they are more similar to each other than to patients from different studies). Treating these nested observations as completely independent would violate basic statistical assumptions, artificially narrowing our confidence intervals and increasing the risk of false-positive discoveries (Type I errors).

To correct for this study-level clustering without losing patient-level granularity, we employed Robust Variance Estimation (RVE) with Satterthwaite degrees-of-freedom adjustments. Conceptually, RVE functions as a statistical safeguard that adjusts the standard errors by acknowledging the groupings within the data. This framework guarantees that our pooled estimates and clinical conclusions remains statistically conservative, realistic, and robust, regardless of the internal correlation or the variation in sample sizes across individual reports.

Small study effects and potential reporting biases were visually explored using funnel plots. Funnel plots were constructed using model residuals for the primary analysis of the multisystem burden. Conventional funnel plots based on the observed effect sizes were examined for system-specific analyses. Egger’s regression test was used as an exploratory assessment of funnel plot asymmetry when the number of effect sizes was considered sufficient for cautious interpretation. Statistical significance was set at *p* < 0.05 for all analyses.

This approach was specifically selected to address the hierarchical structure of the evidence base, in which multiple observations were nested within the studies.

## 3. Results

### 3.1. Study Selection

The database search identified 279 records (PubMed, *n* = 248; Embase, *n* = 25; Web of Science, *n* = 5; and Scopus, *n* = 1). After the removal of 14 duplicate records and 18 records deemed irrelevant prior to screening, 247 records underwent title and abstract screening. Of these, 158 were excluded, and 89 full-text articles were retrieved. Twenty-seven reports could not be retrieved, leaving 62 full-text articles for the eligibility assessment.

A total of 37 studies were excluded for the following reasons: studies conducted outside high-income countries (*n* = 12), incomplete or non-extractable data (*n* = 10), reports limited to isolated dental manifestations without sufficient systemic information (*n* = 10), and duplicate populations (*n* = 5). Ultimately, 25 studies [7,8,9,10,15,16,17,18,19,20,22,23,24,25,26,32,33,34,35,36,37,38,39,40,41] were included in both the qualitative synthesis and the multilevel meta-analysis.

**Figure 1:**
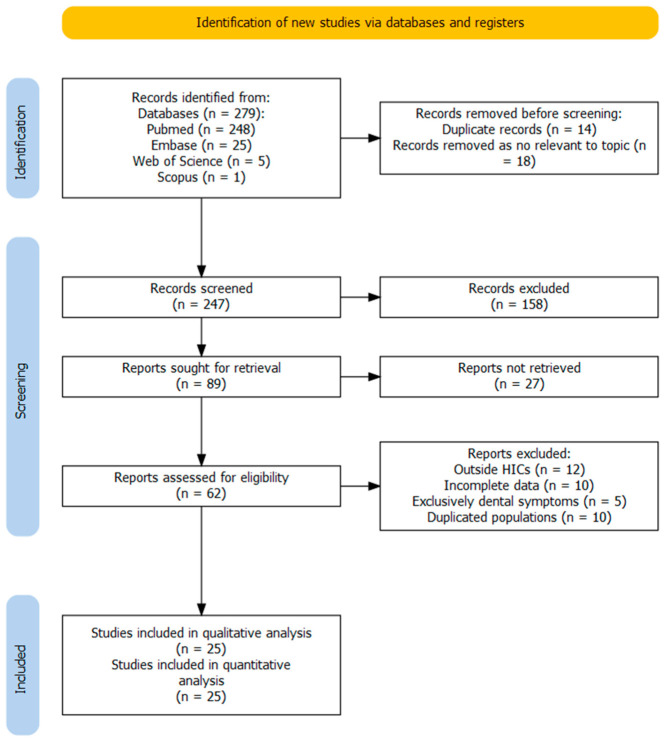
PRISMA 2020 flow diagram of study selection. The figure summarizes the identification, screening, eligibility, and inclusion of studies reporting scurvy in high-income countries between 2012 and 2025. In total, 279 records were identified across four databases; after removal of duplicates and clearly irrelevant records, 247 titles and abstracts were screened, 62 full-text articles were assessed for eligibility, and 25 studies were included in the final synthesis.

The study selection process is illustrated in the PRISMA 2020 flowchart (Figure 1).

### 3.2. Study Characteristics

The included studies (*n* = 25) [7,8,9,10,15,16,17,18,19,20,22,23,24,25,26,32,33,34,35,36,37,38,39,40,41] were geographically concentrated in high-income regions, predominantly North America and Western Europe. The United States contributed the largest number of reports [8,10,16,20,22,24,33,34,35,36,39], followed by France [15,23,32,41]. Additional cases were identified in Australia [17,40], Italy [18,37], Belgium [7], Saudi Arabia [9], Ireland [19], Canada [25], Germany [26], and Puerto Rico [38], all of which were classified as high-income countries according to the World Bank criteria [11].

The evidence base was overwhelmingly pediatric, with the majority of studies reporting cases in children and adolescents [7,8,9,10,15,16,17,18,19,20,23,24,25,26,33,34,37,38,39,40,41], whereas only two older adults were described in a French case series [32]. Reported ages ranged from 18 months in infants and toddlers [15,41] to 87 years in the oldest adult [32].

A comprehensive overview of the sociodemographic and methodological characteristics of the included studies, including the study design, sample size, clinical setting, and contextual risk factors, is presented in Table 1.

### 3.3. Risk-of-Bias Assessment

Overall, the methodological quality varied across study designs, reflecting the heterogeneous and predominantly descriptive nature of the evidence base. Among the 16 case reports [7,8,9,15,17,18,19,20,22,25,26,33,34,36,38,41], most fulfilled key JBI domains related to diagnostic ascertainment, clinical description, intervention, and outcomes, with only a few items rated as unclear or incomplete.

The four case series showed greater variability in the results. One radiology-based pediatric series [10] was judged to have great methodological concern due to unclear inclusion criteria, incomplete case recruitment, and limited reporting of co-interventions and follow-up data. The remaining three case series were considered to have moderate methodological limitations, primarily related to the retrospective design and incomplete reporting of confounders [23,24,32].

All five observational cohort studies [16,35,37,39,40] were rated as having an overall moderate risk of bias using ROBINS-I, mainly driven by residual confounding and missing data, despite the low risk in domains related to exposure classification, outcome measurement and deviations from the intended interventions.

Overall, the risk of bias across the included studies was predominantly low to moderate, with higher methodological concerns concentrated in case series designs and driven primarily by limitations in study design, reporting completeness, and control of confounding factors.

**Figure 2:**
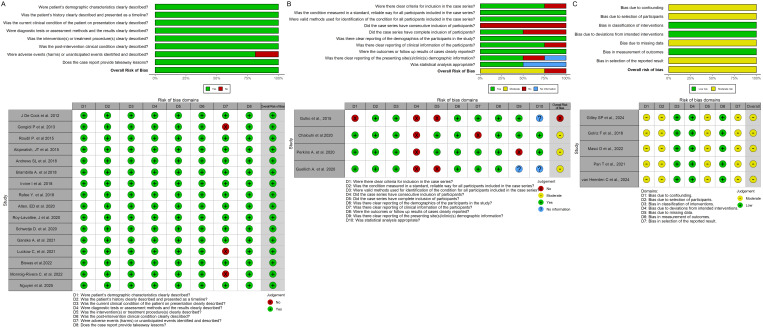
Risk-of-bias assessment across study designs [7,8,9,10,15,16,17,18,19,20,22,23,24,25,26,32,33,34,35,36,37,38,39,40,41]. Panel (**A**) displays the JBI appraisal of case reports, showing generally low methodological concern across domains. Panel (**B**) summarizes the JBI assessment for case series, highlighting moderate variability in methodological rigor. Panel (**C**) presents the ROBINS-I assessment of observational studies, indicating an overall moderate risk of bias driven primarily by confounding and missing data.

A graphical synthesis of the risk-of-bias assessment across the study designs is shown in Figure 2.

### 3.4. Meta-Analysis Results

The multilevel random-effects meta-analysis of multisystem involvement showed a pooled mean of 2.29 affected organ systems per patient (95% CI: 1.62–2.96), indicating that scurvy typically presents as a multisystem condition involving more than two clinical domains simultaneously.

Between-study heterogeneity was substantial (*I*^2^ = 66.8%, τ^2^ = 1.2001, *p* < 0.001), reflecting variability in clinical presentation, case mix, and reporting practices. The prediction interval (0.00–4.68) suggests considerable dispersion of the expected values across comparable populations.

**Figure 3:**
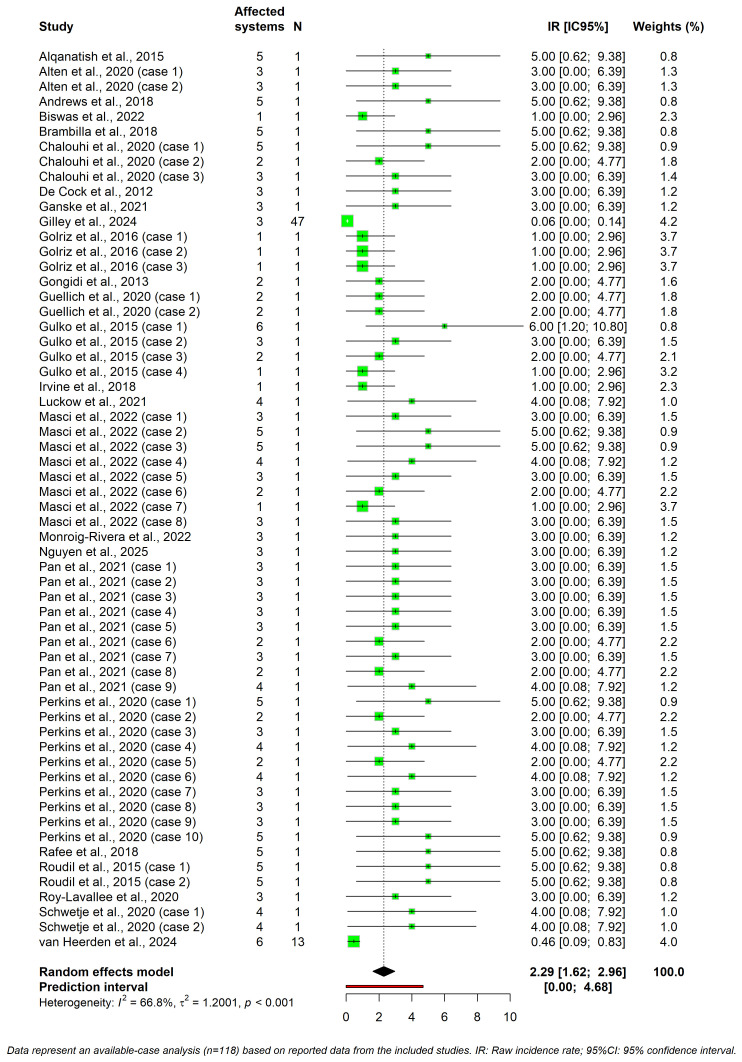
Multilevel random-effects meta-analysis of multisystem involvement in scurvy [7,8,9,10,15,16,17,18,19,20,22,23,24,25,26,32,33,34,35,36,37,38,39,40,41]. Each square represents the estimated number of affected systems for individual cases or study subgroups, with horizontal lines indicating 95% confidence intervals. The diamond represents the pooled estimate and its confidence interval, with the prediction interval reflecting expected variability in future populations. Note: The evidence base predominantly comprised pediatric patients, with limited representation of older adults. Most cases arose in clinically selected and vulnerable populations characterized by restrictive dietary patterns, neurodevelopmental or behavioral conditions, medical complexity, or other nutritional and psychosocial risk factors. Accordingly, the pooled estimate should not be interpreted as representative of the general pediatric, geriatric, or overall population.

These findings are illustrated in Figure 3.

System-specific analyses (Table 2) showed that musculoskeletal involvement had the highest estimated odds among the evaluated clinical domains (OR 1.82; 95% CI: 0.98–3.38), whereas neurological manifestations were significantly less frequent (OR 0.42; 95% CI: 0.28–0.62; *p* < 0.001). Cutaneous, gingival/mucosal, hematological, and systemic manifestations showed intermediate prevalence with overlapping confidence intervals.

Odds ratios, heterogeneity indices (*I*^2^, τ^2^), Cochran’s Q, Z-statistics, and Egger’s test were presented for each clinical domain.

Multivariable meta-regression analysis (Table 3) identified data source type (individual vs. aggregate) as a statistically significant predictor of the number of affected systems (IRR 4.64; *p* = 0.045), whereas other candidate moderators, including age, restrictive diet, neurodevelopmental disorders, biochemical confirmation, diagnostic imaging, time to diagnosis, and hospital size, showed no consistent associations.

The data source type (individual vs. aggregate) was significantly associated with an increased number of affected systems (IRR 4.64; *p* = 0.045).

Further system-specific multivariable models (Table 4) indicated that age was the only significant moderator of musculoskeletal involvement (OR 0.97; *p* = 0.035), suggesting a slight decrease in musculoskeletal manifestations with increasing age.

Age was the only statistically significant moderator of musculoskeletal involvement (OR 0.97; *p* = 0.035), whereas other variables showed no consistent associations.

### 3.5. Small-Study Effects and Publication Bias

Although Egger’s regression tests were statistically significant across the system-specific analyses, visual inspection of the funnel plots did not reveal pronounced asymmetry. Given the limited number of contributing studies, the predominance of case-based evidence, and the known limitations of asymmetry tests in small and heterogeneous meta-analyses, these findings should be interpreted cautiously and not considered definitive evidence of publication bias.Residual-based funnel plots for the primary model and system-specific diagnostic plots are provided in the Supplementary Material (Figures S1 and S2). Owing to the limited number of contributing studies and heterogeneity of the evidence base, the formal interpretation of funnel plot symmetry should be approached with caution.

### 3.6. Clinical Characteristics

#### 3.6.1. Demographics and Risk Factors

Several recurrent contextual risk factors have been identified in previous studies. Food insecurity, social deprivation, and homelessness have been reported in pediatric and young adult populations [16,26,37,39,40]. Neurodevelopmental disorders, particularly autism spectrum disorder, were reported in several cases [7,8,17,20,34], whereas extreme food selectivity or restrictive dietary patterns were also frequently described [10,18,23,24,35,36,40].

Restrictive dietary patterns, including highly selective eating behaviors, eating disorders, dietary restriction, and dietary deficiency, have been consistently reported [10,16,18,23,25,37,38,39].

Additional clinical contexts included oncological treatment [19], chronic gastrointestinal disease or malabsorption [16,37], and advanced age combined with social isolation [32].

#### 3.6.2. Clinical Presentation

Scurvy consistently presents as a multisystem condition involving several organ systems. Musculoskeletal symptoms were particularly prominent and frequently represented the primary reason for clinical evaluation [9,10,15,17,18,19,22,23,35,36,37,38,40].

Cutaneous and mucosal manifestations, including perifollicular hemorrhage, purpura, petechiae, ecchymoses, and gingival bleeding, were commonly reported across age groups [7,15,16,18,19,24,26,32,37,38,39,41].

Hematological abnormalities (mainly anemia) and systemic features, such as fatigue, irritability, and poor growth, are also frequent [15,16,19,26,32,33,37,38,39,40,41].

Neurological manifestations are comparatively uncommon and typically associated with complex or chronic clinical contexts [9,20,26], as detailed in Table 2.

#### 3.6.3. Diagnostic Approaches

Diagnosis was most commonly established through a combination of clinical suspicion and biochemical confirmation of low serum vitamin C levels (<11 µmol/L) [9,15,16,19,26,37,40]. In some cases, the diagnosis was based on clinical presentation and response to treatment when laboratory confirmation was unavailable [7,15,24,32].

Diagnostic delay is frequent, often ranging from several weeks to months, with reported median delays of approximately 12 weeks [9,10,18,25,39,40]. These delays were particularly pronounced in patients with atypical presentations or complex comorbidities [9,18,26,39].

Extensive diagnostic workups, including imaging and invasive procedures, were frequently performed before scurvy was considered [10,15,17,23,35,36,38,40].

#### 3.6.4. Nutritional Management and Outcomes

All patients received vitamin C supplementation, administered orally or intravenously, depending on clinical severity [7,9,10,16,17,18,19,23,25,26,32,33,37,38,39,40,41]. The reported doses ranged from 100 to 1000 mg/day.

Clinical improvement was typically rapid, with symptom relief often observed within 2–4 weeks [7,9,10,17,18,19,23,26,32,37,38,39,40,41] and complete resolution usually achieved within 3–6 months [7,16,17,18,19,23,25,26,32,33,37,38,39,40,41].

Relapse is uncommon and is generally associated with poor adherence to or persistence of underlying risk factors [19,25,38,39,40,41].

### 3.7. Geographic Distribution

The included studies were primarily concentrated in North America and Western Europe, with additional contributions from Australia [17,40], Italy [18,37], Belgium [7], Saudi Arabia [9], Canada [25], Germany [26], and Puerto Rico [38]. All countries met the World Bank’s definition of high-income settings [11].

The geographic and economic context of the included studies is summarized in Table 5.

This table summarizes the country-level data, including the World Bank classification, geographic coordinates, and economic indicators for each study.

No studies were identified from Nordic high-income countries, Eastern Europe, or high-income Asian regions, such as Japan or South Korea, suggesting potential underreporting or publication gaps in these regions.

## 4. Discussion

### 4.1. Principal Findings and Conceptual Interpretation

This systematic review and meta-analysis provide the most comprehensive synthesis to date of contemporary scurvy cases in high-income countries (HICs; World Bank classification) between 2012 and 2025. Contrary to the long-standing perception of scurvy as a historically eradicated disease, our findings demonstrate that it persists as a clinically relevant and under-recognized condition in modern healthcare systems.

Quantitative synthesis adds a novel dimension to our understanding of this condition. The multilevel meta-analysis showed that patients diagnosed with scurvy frequently presented with involvement of multiple organ systems involving a mean of 2.29 organ systems per patient (95% CI: 1.62–2.96), reinforcing that its clinical expression extends beyond isolated and classical manifestations. This finding challenges the traditional reductionist view of scurvy as primarily a mucocutaneous disorder and supports a broader systemic conceptualization.

Importantly, disease burden is not randomly distributed across the population. Instead, it is concentrated in specific vulnerability clusters, particularly among children with neurodevelopmental disorders, individuals with restrictive dietary patterns, and socially marginalized populations [6,8,9,10,15,16,17,18,19,25,33,39]. These patterns suggest that the persistence of scurvy in HICs may be associated with structural and behavioral factors influencing nutritional intake within otherwise resource-rich environments. This pattern aligns with the concept of “hidden hunger,” in which micronutrient deficiencies persist despite apparent caloric sufficiency.

However, the available evidence does not allow attribution of all multisystem manifestations exclusively to vitamin C deficiency. Several included studies reported limited nutritional characterization, and concomitant deficiencies of other micronutrients, including iron, zinc, vitamin D, vitamin A, and other fat-soluble vitamins, may have contributed to the observed clinical presentations. Therefore, the multisystem burden described in this review should be interpreted within the broader context of complex nutritional vulnerability rather than as a direct consequence of isolated vitamin C deficiency alone.

Therefore, the persistence of contemporary scurvy in high-income settings appears to emerge from a complex interaction between restrictive dietary behaviors, social vulnerability, insufficient nutritional surveillance, and delayed clinical recognition of micronutrient deficiencies.

### 4.2. A Shift in Epidemiological Paradigm

One of the most relevant contributions of this review is the identification of a shift in the epidemiological profile of scurvy in the past decade. Historically associated with prolonged deprivation and maritime isolation, contemporary scurvy in HICs emerges in a fundamentally different context.

Current cases were most frequently reported in the context of:

- Neurodevelopmental conditions (e.g., autism spectrum disorder)
- Extreme dietary selectivity or restrictive eating behaviors
- Psychiatric disorders and social vulnerability
- Complex chronic disease contexts (oncological, gastrointestinal) [13,14,15,16,17]

This transition reflects a shift from macro-level deprivation to micro-level nutritional exclusion, where adequate food availability coexists with inadequate food intake. In this sense, modern scurvy may reflect patterns of selective malnutrition rather than absolute scarcity of food. From a public health perspective, this pattern aligns with the concept of “hidden hunger,” wherein micronutrient deficiencies persist despite adequate calorie intake. This paradox highlights the need for targeted nutritional surveillance strategies in high-income settings, particularly among populations with behavioral or clinical risk profiles.

Although detailed dietary histories have been inconsistently reported across studies, restrictive and highly selective eating patterns have emerged as one of the most recurrent contextual risk factors associated with contemporary scurvy presentations.

Importantly, most studies did not systematically assess overall nutritional status or the presence of additional micronutrient deficiencies. Consequently, vitamin C deficiency may represent one component of a broader pattern of nutritional inadequacy in these vulnerable populations.

Furthermore, the strong pediatric predominance observed in the included studies suggests that developmental and behavioral factors play central roles in contemporary risk profiles. This is consistent with previous observations highlighting selective eating patterns in children with autism and other neurodevelopmental conditions [13,14].

### 4.3. Clinical Complexity and Diagnostic Delay

The clinical heterogeneity identified in this review represents a major barrier to the timely diagnosis of this condition. While classical features remain, a substantial proportion of cases exhibit atypical or nonspecific manifestations, including musculoskeletal pain, limping, fatigue, irritability, and inflammatory-like presentations [10,18,20,25,34,37,39].

This variability contributes directly to diagnostic delays, which frequently extend for weeks or months [9,10,18,25,39,40]. In many cases, scurvy was only considered after extensive diagnostic workups, including imaging and invasive procedures, failed to provide an alternative explanation [10,15,17,23,35,36,38,40]. Although the overall heterogeneity was relatively high (*I*^2^ = 66.8%), our multivariable meta-regression framework (Table 2 and Table 3) demonstrated that this variance could not be attributed to specific diagnostic criteria or geographical settings, confirming that the clinical expression of contemporary pediatric scurvy remains highly consistent across different high-risk vulnerability profiles.

From a clinical reasoning perspective, this reflects a diagnostic blind spot in modern medicine. The assumption that micronutrient deficiencies are unlikely in HICs leads to the systematic exclusion of nutritional etiologies from the initial differential diagnosis. This phenomenon is particularly evident in patients with complex clinical backgrounds, in whom symptoms are often attributed to underlying chronic conditions rather than nutritional deficiency.

Failure to consider scurvy early in the diagnostic process contributes to unnecessary investigations and prolonged morbidity.

### 4.4. Integration of Quantitative Findings

The meta-analytic results provide important insights into the structure of clinical involvement. The predominance of musculoskeletal manifestations (OR 1.82; 95% CI: 0.98–3.38) aligns with the high frequency of pain, limping, and functional impairment in patients with scurvy. Conversely, the significantly lower prevalence of neurological involvement (OR 0.42; 95% CI: 0.28–0.62; *p* < 0.001) suggests that central nervous system manifestations are not the primary drivers of clinical suspicion.

The identification of age as a moderator of musculoskeletal involvement (OR 0.97; *p* = 0.035) indicates a subtle but relevant age-related gradient, potentially reflecting differences in skeletal metabolism, growth dynamics, and reporting patterns across different age groups.

Additionally, the finding that the data source type significantly influenced multisystem involvement (IRR 4.64; *p* = 0.045) highlights an important methodological consideration. Studies based on individual case reports tend to capture more extensive clinical details, whereas aggregated cohort data may underestimate the complexity. This reinforces the importance of using multilevel models that can account for the hierarchical data structures.

### 4.5. Treatment Response and Health System Implications

Despite the diagnostic challenges, treatment outcomes were consistently favorable. Vitamin C supplementation and dietary modification were consistently associated with rapid clinical improvement, typically within 2–4 weeks, with complete recovery in most cases within a few months [7,16,17,18,19,23,25,26,32,33,37,38,39,40,41].

However, the occurrence of relapse in a subset of patients underscores the fact that the effectiveness of nutritional management is contingent on addressing underlying risk factors. Nutritional supplementation alone may not be sufficient in the presence of persistent behavioral, psychiatric, or socioeconomic determinants.

In many cases, long-term prevention requires sustained dietary interventions, caregiver education, and multidisciplinary nutritional support to correct restrictive or highly selective eating behaviors.

From a health systems perspective, the available evidence suggests that contemporary scurvy frequently occurs within multidimensional clinical and nutritional vulnerability contexts, potentially benefiting from integrated approaches involving the following:

- Clinical management
- Nutritional intervention
- Behavioral and psychological support
- Social and environmental assessment

From a clinical standpoint, scurvy should be actively screened for in patients with unexplained musculoskeletal symptoms and restrictive dietary patterns.

However, standardized dietary intervention protocols and long-term nutritional management strategies have been inconsistently reported across studies, limiting the ability to formulate specific evidence-based management recommendations.

### 4.6. Strengths and Methodological Contributions

This study had several methodological strengths that should be noted. To our knowledge, this is the first systematic review to combine qualitative synthesis with multilevel meta-analytic modeling in the context of scurvy. The use of robust variance estimation allowed for the inclusion of heterogeneous data structures, including clustered case reports and case series, without violating statistical assumptions. This approach allows the integration of hierarchically structured evidence, which is typically excluded from conventional meta-analyses.

Furthermore, the operationalization of scurvy as a multisystem burden (number of affected systems) represents an innovative approach that moves beyond binary or single-outcome analyses. This framework may be applicable to other conditions characterized by heterogeneous clinical features.

The rigorous application of the PRISMA 2020 guidelines and validated risk-of-bias tools (JBI and ROBINS-I) further strengthened the transparency and reproducibility of the findings.

### 4.7. Limitations

This study has several limitations that must be considered. First, the evidence base is dominated by pediatric case reports and small case series involving vulnerable patients with pre-existing conditions, which introduce potential publication bias and limit generalizability. Second, the relatively small number of studies contributing to the quantitative synthesis resulted in substantial heterogeneity (*I*^2^ = 66.8%), reflecting both clinical and methodological variations.

Third, inconsistencies in reporting, particularly regarding diagnostic criteria, laboratory thresholds, and outcome definitions, limited the ability to perform more granular subgroup analyses. Finally, the absence of data from certain high-income regions suggests potential underreporting or publication bias. Additionally, detailed dietary intake assessments, standardized nutritional surveillance data, biochemical nutritional profiling, and structured dietary intervention protocols were inconsistently reported across the included studies, limiting causal interpretation and restricting the ability to derive evidence-based nutritional management recommendations. Importantly, most studies did not systematically evaluate coexisting micronutrient deficiencies, such as iron, zinc, vitamin D, vitamin A, or other fat-soluble vitamins. Therefore, the extent to which the reported multisystem manifestations were attributable exclusively to vitamin C deficiency cannot be fully established. This limitation should be considered when interpreting the pooled estimates of multisystem involvement.

### 4.8. Implications for Clinical Practice and Research

The findings of this review have direct implications for clinical practice. Scurvy should be considered in the differential diagnosis of patients presenting with unexplained multisystem symptoms, particularly when risk factors such as restrictive diets, neurodevelopmental disorders, or social vulnerability are present.

Routine dietary assessment and targeted vitamin C screening may be low-cost, high-impact strategies for reducing diagnostic delays. Targeted screening should be considered, particularly in high-risk groups, including patients with autism spectrum disorder, eating disorders, oncological conditions, and restrictive dietary patterns. Particular clinical attention should be given to patients presenting with restrictive dietary behaviors, unexplained musculoskeletal symptoms, neurodevelopmental disorders, recurrent fatigue, mucocutaneous manifestations, or delayed wound healing, especially when multiple features coexist. Clinicians should maintain a high index of suspicion in cases of unexplained musculoskeletal pain accompanied by dietary restrictions. When clinically suspected, early dietary assessment and plasma vitamin C testing may facilitate prompt diagnosis and prevent unnecessary invasive investigations. Increased awareness among clinicians is essential to prevent unnecessary investigations and prolonged morbidity.

Future research should prioritize the following areas:

- Prospective cohort studies in at-risk populations
- Standardization of diagnostic criteria and reporting
- Integration of nutritional screening into routine care pathways
- Evaluation of long-term outcomes and relapse prevention strategies

### 4.9. Comparison with the Previous Literature

The previous literature on scurvy in high-income settings has been largely limited to narrative reviews and isolated case reports [21]. While earlier studies have highlighted the persistence of nutritional deficiencies in specific populations [13,14], none have systematically quantified the clinical burden or explored its determinants using advanced analytical methods.

This study extends existing knowledge by providing a comprehensive synthesis and quantitative framework, demonstrating that contemporary scurvy is not merely a sporadic anomaly but a structured phenomenon linked to identifiable risk profiles.

## 5. Conclusions

Scurvy remains a clinically relevant condition in high-income countries, predominantly affecting vulnerable populations characterized by restrictive dietary patterns, neurodevelopmental disorders, and social vulnerability. Far from being a historical curiosity, it continues to occur within modern healthcare systems and may represent an important indicator of underlying nutritional vulnerability.

This review suggests that contemporary scurvy is frequently associated with multisystem manifestations and heterogeneous clinical presentations, often contributing to diagnostic delays and unnecessary investigations. Nevertheless, the condition remains highly responsive to vitamin C supplementation, with most reported cases showing rapid clinical improvement and favorable recovery following appropriate nutritional management.

The findings underscore the importance of maintaining clinical suspicion for nutritional deficiencies in patients presenting with unexplained musculoskeletal, mucocutaneous, or systemic symptoms, particularly when restrictive dietary behaviors or other vulnerability factors are present. However, because nutritional assessments and comprehensive micronutrient evaluations were inconsistently reported across the included studies, the contribution of concomitant nutritional deficiencies cannot be excluded and warrants further investigation.

Future prospective studies incorporating standardized dietary assessments and broader nutritional profiling are needed to better characterize the determinants, clinical spectrum, and prevention strategies associated with contemporary scurvy in high-income settings.

## Figures and Tables

**Table 1:** Sociodemographic and methodological characteristics of the included studies.

Author	Year	Country	Study Design	Age Range	Sample Size	Setting	Population Risk Context
De Cock [7]	2012	Belgium	Case Report	3 y	1	Radiology Unit	Autism
Gongidi [8]	2013	United States	Case Report	5 y	1	Pediatric Radiology	Autism
Alqanatish [9]	2015	Saudi Arabia	Case Report	12 y	1	Pediatrics	Food insecurity
Gulko [10]	2015	United States	Case Series	5–8 y	4	Radiology	Selective eating
Roudil [15]	2015	France	Case Reports	1.5–3 y	2	Dermatology	Neglect
Golriz [16]	2017	United States	Retrospective Study	3–17 y	32	Radiology	Eating disorder
Andrews [17]	2018	Australia	Case Report	6 y	1	Pediatrics	Autism
Brambilla [18]	2018	Italy	Case Report	3 y	1	Pediatrics	Dietary restriction
Irvine [19]	2019	Ireland	Case Report	2 y	1	Oncology	Cancer treatment
Rafee [20]	2019	United States	Case Report	14 y	1	Pediatrics	Autism
Alten [22]	2020	United States	Case Report	5–12 y	2	Endocrinology	Bone health
Chalouhi [23]	2020	France	Case Series	3–3.5 y	3	Pediatrics	Restricted diet
Guellich [32]	2021	France	Case Series	69–87 y	2	Internal Medicine	Social isolation
Perkins [24]	2020	United States	Case Series	3–20 y	10	Pediatrics	Developmental delay
Roy-Lavallée [25]	2020	Canada	Case Report	16 y	1	Adolescent Medicine	Anorexia nervosa
Schwetje [26]	2020	Germany	Case Reports	6 y	2	Pediatrics	Malnutrition
Ganske [33]	2021	United States	Case Report	9 y	1	Radiology	Limp
Luckow [34]	2021	United States	Case Report	5 y	1	Emergency Dept	Autism
Pan [35]	2021	United States	Retrospective study	3–13 y	9	Orthopedics	Orthopedic case
Biswas [36]	2022	United States	Case Report	6 y	1	Radiology	Atypical presentation
Masci [37]	2022	Italy	Observational Study	1–10 y	8	Pediatrics	Hospitalized children
Monroig-Rivera [38]	2022	Puerto Rico	Case Report	2 y	1	Pediatrics	Dietary deficiency
Gilley [39]	2024	United States	Retrospective study	3–12 y	47	Hospital-based	Musculoskeletal
Van Heerden [40]	2024	Australia	Observational Study	0–18 y	13	Population-based	National registry
Nguyen [41]	2025	France	Case Report	18 m	1	Pediatrics	Misdiagnosed as rickets

HICs = High-Income Countries. This table summarizes the basic characteristics of the 25 studies included in the qualitative synthesis, including geographical distribution, study design (case report, case series, observational), patient demographics, clinical settings, and major risk factors, when available. The sample size refers to individual patients or full cohorts in observational studies.

**Table 2:** Prevalence analysis of clinical manifestations by system.

	Crude Prevalence, *n* (%)	OR (95% CI)	*I* ^2^	τ^2^	Q Test (^a^ *p* Value)	Z Score (^a^ *p* Value)	Egger’s Test (^a^ *p* Value)
Musculoskeletal	116 (98.3%)	1.82 (0.98, 3.38)	23.26%	0.770	0.994	Z = 2.034, *p* = 0.056	<0.001
Cutaneous	88 (74.6%)	0.72 (0.42, 1.23)	11.44%	0.328	0.923	Z = −1.29, *p* = 0.215	<0.001
Gingival mucosal	88 (74.6%)	0.73 (0.44, 1.24)	9.93%	0.280	0.923	Z = −1.256, *p* = 0.227	<0.001
Hematological	48 (40.7%)	0.83 (0.44, 1.56)	23.64%	0.802	0.952	Z = −0.616, *p* = 0.545	0.007
Systemic	36 (30.5%)	0.69 (0.4, 1.19)	11.74%	0.345	0.983	Z = −1.424, *p* = 0.173	0.009
Neurological	23 (19.5%)	0.42 (0.28, 0.62)	0%	0.000	>0.999	Z = −4.914, *p* < 0.001	0.027

Data represent an available-case analysis (*n* = 118) based on reported data from the included studies. OR: Odds ratio; 95%CI: 95% confidence interval; Q: Cochran’s Q test. ^a^ significant if *p* < 0.05 (shown in red).

**Table 3:** Multivariable meta-regression of factors associated with multisystem involvement.

	IRR (95% CI)	^a^ *p* Value
(Intercept)	30.1 (1.19, 762.55)	0.041
Age	0.99 (0.93, 1.04)	0.513
Restrictive diet (yes)	1.75 (0.12, 25.92)	0.604
Neurodevelopmental disorder (yes)	0.84 (0.42, 1.7)	0.582
Vitamin C laboratory diagnosis (<11 µmol/L) (yes)	0.23 (0.04, 1.36)	0.083
Diagnostic imaging (X-ray/MRI) (yes)	0.39 (0.11, 1.39)	0.112
Time before diagnosis weeks	0.99 (0.97, 1.02)	0.373
Data source type (individual/case-based)	4.64 (1.05, 20.63)	0.045
Hospital range (small/medium (<500 beds))	0.81 (0.21, 3.15)	0.733

IRR: Incidence rate ratio; 95% CI: 95% confidence interval. ^a^ significant if *p* < 0.05 (shown in red).

**Table 4:** Multivariable meta-regression analysis of factors associated with clinical manifestations in scurvy.

	Musculoskeletal		Cutaneous		Gingival Mucosal		Hematological		Systemic		Neurological	
	OR (95% CI)	^a^ *p* Value	OR (95% CI)	^a^ *p* Value	OR (95% CI)	^a^ *p* Value	OR (95% CI)	^a^ *p* Value	OR (95% CI)	^a^ *p* Value	OR (95% CI)	^a^ *p* Value
(Intercept)	1.17 (0.11, 12.61)	0.883	0.85 (0.03, 21.9)	0.913	0.58 (0.05, 7.35)	0.634	0.26 (0, 14.79)	0.472	2.68 (0.15, 46.49)	0.451	3.36 (0.41, 27.31)	0.218
Data source type (individual/case-based)	3.64 (0.4, 33.25)	0.213	3.04 (0.59, 15.76)	0.155	3.73 (0.82, 16.85)	0.078	3.45 (0.45, 26.52)	0.195	2.12 (0.32, 13.99)	0.376	1.06 (0.3, 3.73)	0.916
Diagnostic imaging (X-ray/MRI) (yes)	0.82 (0.45, 1.5)	0.444	0.47 (0.11, 2)	0.243	0.76 (0.22, 2.55)	0.584	1.03 (0.16, 6.82)	0.966	0.45 (0.1, 2.1)	0.246	0.65 (0.2, 2.15)	0.401
Vitamin C laboratory diagnosis (<11 µmol/L) (yes)	0.98 (0.6, 1.62)	0.934	0.41 (0.16, 1.03)	0.056	0.71 (0.17, 2.87)	0.541	1.17 (0.53, 2.57)	0.623	0.91 (0.34, 2.46)	0.818	0.32 (0.09, 1.18)	0.074
Age	0.97 (0.95, 1)	0.035	1.02 (0.96, 1.07)	0.397	0.98 (0.95, 1.01)	0.158	0.99 (0.92, 1.06)	0.687	0.99 (0.95, 1.03)	0.586	0.98 (0.96, 1.01)	0.208
Hospital range (small/medium (<500 beds))	1.02 (0.25, 4.09)	0.976	0.93 (0.29, 2.95)	0.886	1.06 (0.32, 3.5)	0.914	0.62 (0.17, 2.28)	0.443	1.14 (0.27, 4.75)	0.844	1.67 (0.57, 4.86)	0.304
Neurodevelopmental disorder (yes)	0.86 (0.55, 1.35)	0.470	0.67 (0.3, 1.46)	0.269	1.06 (0.61, 1.85)	0.819	0.52 (0.25, 1.1)	0.078	0.73 (0.35, 1.52)	0.357	0.77 (0.42, 1.41)	0.364
Restrictive diet (yes)	1.1 (0.6, 2.04)	0.702	2.52 (0.53, 12.07)	0.188	1.04 (0.15, 7.43)	0.960	2.6 (0.42, 16.17)	0.240	0.37 (0.06, 2.34)	0.222	0.51 (0.1, 2.67)	0.333
Time before diagnosis weeks	1 (0.99, 1.02)	0.530	0.99 (0.98, 1)	0.059	1 (0.98, 1.03)	0.743	1 (0.99, 1.02)	0.231	1 (0.98, 1.01)	0.286	1 (0.95, 1.04)	0.545
Global statistics												
	*I*^2^: 25.09%	^a^ QM *p*-value: 0.955	*I*^2^: 11.59%	^a^ QM *p*-value: 0.748	*I*^2^: 13.59%	^a^ QM *p*-value: 0.880	*I*^2^: 24.37%	^a^ QM *p*-value: 0.881	*I*^2^: 13.26%	^a^ QM *p*-value: 0.898	*I*^2^: 0%	^a^ QM *p*-value: 0.992

OR: Odds ratio; 95% CI: 95% confidence interval; QM: Test of Moderators. ^a^ significant if *p* < 0.05 (shown in red).

**Table 5:** Geographic and economic characteristics of included studies.

Author	Year	Country	Income Classification (WB)	Latitude	Longitude	GNI per Capita (Thousands of Current USD)
De Cock [7]	2012	Belgium	High	50.85	4.35	48.08
Gongidi [8]	2013	United States	High	~28.54	−81.38	76.37
Alqanatish [9]	2015	Saudi Arabia	High	24.71	46.67	27.941
Gulko [10]	2015	United States	High	40.71	−74.01	76.37
Roudil [15]	2015	France	High	48.85	2.35	44.04
Golriz [16]	2017	United States	High	~29.76	−95.37	76.37
Andrews [17]	2018	Australia	High	−37.81	144.96	62.43
Brambilla [18]	2018	Italy	High	43.77	11.25	37.45
Irvine [19]	2019	Ireland	High	~53.33	−6.32	72
Rafee [20]	2019	United States	High	~43.01	−83.69	76.37
Alten [22]	2020	United States	High	44.95	−93.09	76.37
Chalouhi [23]	2020	France	High	48.85	2.35	44.04
Guellich [32]	2021	France	High	48.85	2.35	44.04
Perkins [24]	2020	United States	High	45.52	−122.67	76.37
Roy-Lavallée [25]	2020	Canada	High	43.65	−79.38	52.96
Schwetje [26]	2020	Germany	High	50.11	8.68	55.56
Ganske [33]	2021	United States	High	44.96	−93.27	76.37
Luckow [34]	2021	United States	High	47.61	−122.32	76.37
Pan [35]	2021	United States	High	40.27	−76.88	76.37
Biswas [36]	2022	United States	High	40.77	−73.96	76.37
Masci [37]	2022	Italy	High	43.77	11.25	37.45
Monroig-Rivera [38]	2022	Puerto Rico (US)	High	18.46	−66.11	22.05
Gilley [39]	2024	United States	High	39.74	−104.99	76.37
Van Heerden [40]	2024	Australia	High	−33.87	151.21	62.43
Nguyen [41]	2025	France	High	48.85	2.35	44.04

WB = World Bank. GNI per capita (USD) values correspond to 2023 World Bank estimates. The latitude and longitude were approximated to the main urban center where each study was conducted. All countries included in this table are classified as high income according to the World Bank threshold of >$13,845 (2023).

## Data Availability

All data generated or analyzed during this study are included in this published article and its Supplementary Material. No new datasets were generated.

## Supplementary Materials

The following supporting information can be downloaded at https://www.mdpi.com/article/10.3390/nu18142213/s1, Figure S1: Residual-based funnel plot for the multilevel model; Figure S2: Diagnostic funnel plots for publication bias assessment across clinical systems.

## Abbreviations

The following abbreviations are used in this manuscript:

CI	Confidence Interval
GNI	Gross National Income
HICs	High-Income Countries
JBI	Joanna Briggs Institute
PRISMA	Preferred Reporting Items for Systematic Reviews and Meta-Analyses
PROSPERO	International Prospective Register of Systematic Reviews
ROBINS-I	Risk of Bias in Non-randomized Studies of Interventions
SE	Standard Error
USD	United States Dollar

## References

[1] Carpenter K.J. (1988). The History of Scurvy and Vitamin C.

[2] Hirschmann J.V., Raugi G.J. (1999). Adult scurvy. J. Am. Acad. Dermatol..

[3] Lind J. (1753). A Treatise of the Scurvy.

[4] Weinstein M., Babyn P., Zlotkin S. (2001). An orange a day keeps the doctor away: Scurvy in the year 2000. Pediatrics.

[5] Institute of Medicine (US) Panel on Dietary Antioxidants and Related Compounds (2000). Dietary Reference Intakes for Vitamin C, Vitamin E, Selenium, and Carotenoids.

[6] Agarwal A., Shaharyar A., Kumar A., Bhat M.S., Mishra M. (2015). Scurvy in pediatric age group—A disease often forgotten?. J. Clin. Orthop. Trauma.

[7] De Cock J., Renard M., Smet M., Breysem L. (2012). Scurvy in a 3-year-old boy: MRI features. J. Belg. Soc. Radiol..

[8] Gongidi P., Johnson C., Dinan D. (2013). Scurvy in an autistic child: MRI findings. Pediatr. Radiol..

[9] Alqanatish J.T., Alqahtani F., Alsewairi W.M., Al-Kenaizan S. (2015). Childhood scurvy: An unusual cause of refusal to walk in a child. Pediatr. Rheumatol..

[10] Gulko E., Collins L.K., Murphy R.C., Thornhill B.A., Taragin B.H. (2015). MRI findings in pediatric patients with scurvy. Skelet. Radiol..

[11] World Bank World Bank Country and Lending Groups. https://datahelpdesk.worldbank.org/knowledgebase/articles/906519.

[12] Powers C.D., Sternberg M.R., Patel S.B., Pfeiffer C.M., Storandt R.J., Schleicher R.L. (2023). Vitamin C Status of US Adults Assessed as Part of the National Health and Nutrition Examination Survey Remained Unchanged between 2003–2006 and 2017–2018. J. Appl. Lab. Med..

[13] Kinlin L.M., Blanchard A.C., Silver S., Morris S.K. (2016). Vitamin deficiencies in pediatric patients admitted to a tertiary care hospital. Paediatr. Child. Health.

[14] Noble J.M., Mandel A., Patterson M.C. (2007). Scurvy and rickets masked by chronic neurologic illness: Revisiting “ancient” diseases. JAMA Neurol..

[15] Roudil P., Jaffelin C., Gay C., Mory O., Stephan J.-L. (2015). Scorbut chez l’enfant: Deux cas. Ann. Dermatol. Vénéréol..

[16] Golriz F., Donnelly L.F., Devaraj S., Krishnamurthy R. (2017). Modern American scurvy—experience with vitamin C deficiency at a large children’s hospital. Pediatr. Radiol..

[17] Andrews S.L., Iyer S., Rodda C., Fitzgerald J. (2018). Scurvy: A rare cause for limp in a child with autism spectrum disorder. J. Paediatr. Child Health.

[18] Brambilla A., Pizza C., Lasagni D., Lachina L., Resti M., Trapani S. (2018). Pediatric Scurvy: When Contemporary Eating Habits Bring Back the Past. Front. Pediatr..

[19] Irvine I., Walshe T., Capra M., Hayes R. (2019). Scurvy: An unusual complication of paediatric cancer treatment. Skelet. Radiol..

[20] Rafee Y., Burrell K., Cederna-Meko C. (2019). Lessons in early identification and treatment from a case of disabling vitamin C deficiency in a child with autism spectrum disorder. Int. J. Psychiatry Med..

[21] Léger D. (2008). Scurvy: Reemergence of nutritional deficiencies. Can. Fam. Physician.

[22] Alten E., Chaturvedi A., Cullimore M., Fallon A., Habben L., Hughes I., O’malley N., Rahimi H., Renodin-Mead D., Schmidt B. (2020). No longer a historical ailment: Two cases of childhood scurvy with recommendations for bone health providers. Osteoporos. Int..

[23] Chalouhi C., Nicolas N., Vegas N., Matczak S., El Jurdi H., Boddaert N., Abadie V. (2020). Scurvy: A New Old Cause of Skeletal Pain in Young Children. Front. Pediatr..

[24] Perkins A., Sontheimer C., Otjen J.P., Shenoi S. (2020). Scurvy Masquerading as Juvenile Idiopathic Arthritis or Vasculitis with Elevated Inflammatory Markers: A Case Series. J. Pediatr..

[25] Roy-Lavallée J., Bahrani B., Weinstein M., Katzman D.K. (2020). Scurvy: An Unexpected Nutritional Complication in an Adolescent Female With Anorexia Nervosa. J. Adolesc. Health.

[26] Schwetje D., Zillekens A., Kieback J.-D., Koob S., Placzek R. (2020). Infantile scurvy: Still a relevant differential diagnosis in Western medicine. Nutrition.

[27] Page M.J., McKenzie J.E., Bossuyt P.M., Boutron I., Hoffmann T.C., Mulrow C.D., Shamseer L., Tetzlaff J.M., Akl E.A., Brennan S.E. (2021). The PRISMA 2020 statement: An updated guideline for reporting systematic reviews. BMJ.

[28] Joanna Briggs Institute Critical Appraisal Tools. https://jbi.global/critical-appraisal-tools.

[29] Sterne J.A.C., Hernán M.A., Reeves B.C., Savović J., Berkman N.D., Viswanathan M., Henry D., Altman D.G., Ansari M.T., Boutron I. (2016). ROBINS-I: A tool for assessing risk of bias in non-randomised studies of interventions. BMJ.

[30] Balduzzi S., Rücker G., Schwarzer G. (2019). How to perform a meta-analysis with R: A practical tutorial. Evid. Based Ment. Health.

[31] Sidik K., Jonkman J.N. (2019). A note on the empirical Bayes heterogeneity variance estimator in meta-analysis. Stat. Med..

[32] Guellich A., Tella E., Mahé E. (2021). Ulcères nécrotiques et purpuriques des membres inférieurs révélant un scorbut: À propos de deux observations. La Rev. Médecine Interne.

[33] Ganske A., Kolbe A.B., Thomas K., Hull N. (2021). Pediatric scurvy MRI appearance. Radiol. Case Rep..

[34] Luckow C., Thomas A.A. (2021). Scurvy in a Pediatric Patient With Autism and Limp: A Case Report. J. Emerg. Med..

[35] Pan T.B., Hennrikus E.F., Hennrikus W.L. (2021). Modern Day Scurvy in Pediatric Orthopaedics: A Forgotten Illness. J. Pediatr. Orthop..

[36] Biswas S., Miller S., Cohen H.L. (2022). Scurvy in A Malnourished Child: Atypical Imaging Findings. J. Radiol. Case Rep..

[37] Masci D., Rubino C., Basile M., Indolfi G., Trapani S. (2022). When the limp has a dietary cause: A retrospective study on scurvy in a tertiary Italian pediatric hospital. Front. Pediatr..

[38] Monroig-Rivera A.C., Valentín-Martínez K.C., Portalatín-Pérez E. (2022). Scurvy in a 29-Month-Old Patient Presenting with a Gower Sign. Cureus.

[39] Gilley S.P., Ta A., Pryor W., Roper B., Erickson M., Fenton L.Z., Tchou M.M.J., Cotter J.M., Moore J.M. (2024). What Do We C in Children With Scurvy? A Case Series Focused on Musculoskeletal Symptoms. Hosp. Pediatr..

[40] Van Heerden C., Cheng D.R., McNab S., Burgess R., Russell A., Wang Y., Bleathman F., Maharaj I., Zhang J., Easterbrook M. (2024). Scurvy and vitamin C deficiency in an Australian tertiary children’s hospital. J. Paediatr. Child Health.

[41] Nguyen A.T., Jalon S., Simon A., Jouret M., Le Lorier J., Hentgen V., Dommergues M.-A. (2025). Scurvy in an 18-month-old child mimicking a clinical presentation of rickets. Arch. Pediatr..

