# Supplementary material for: Contemporary Scurvy in Vulnerable Populations in High-Income Countries: A Systematic Review and Multilevel Meta-Analysis
Source: Nutrients. 2026 Jul 8;18(14):2213. doi: 10.3390/nu18142213 (PMC13415094; doi:10.3390/nu18142213)

Supplementary material

Supplementary material. Figure S1: Residual-based funnel plot for the multilevel model.

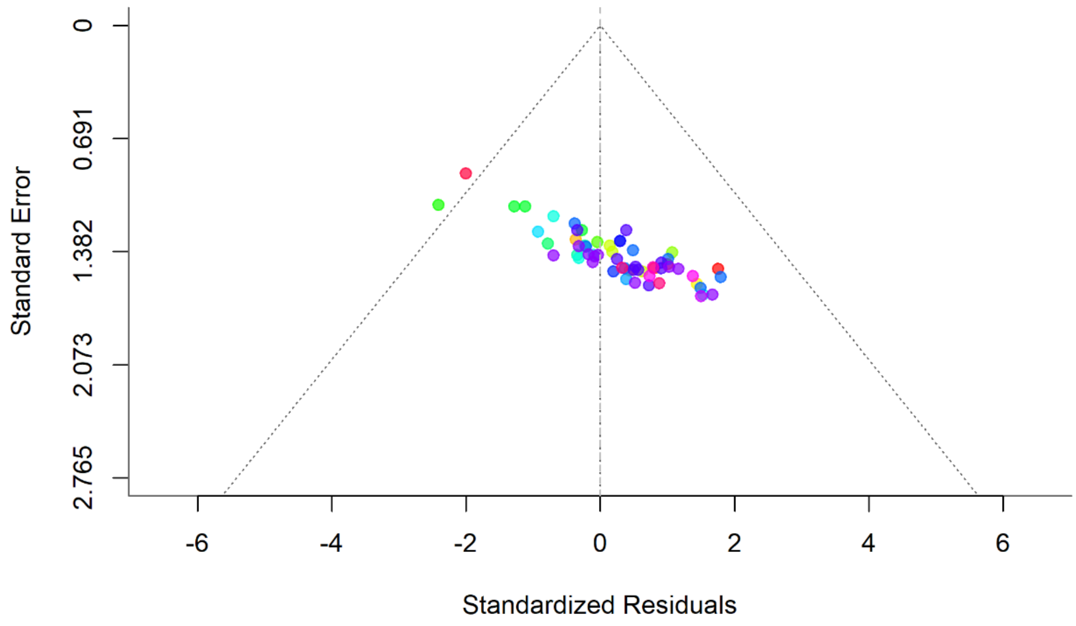

Supplementary material. Figure S2: Diagnostic funnel plots for publication bias assessment across clinical systems.

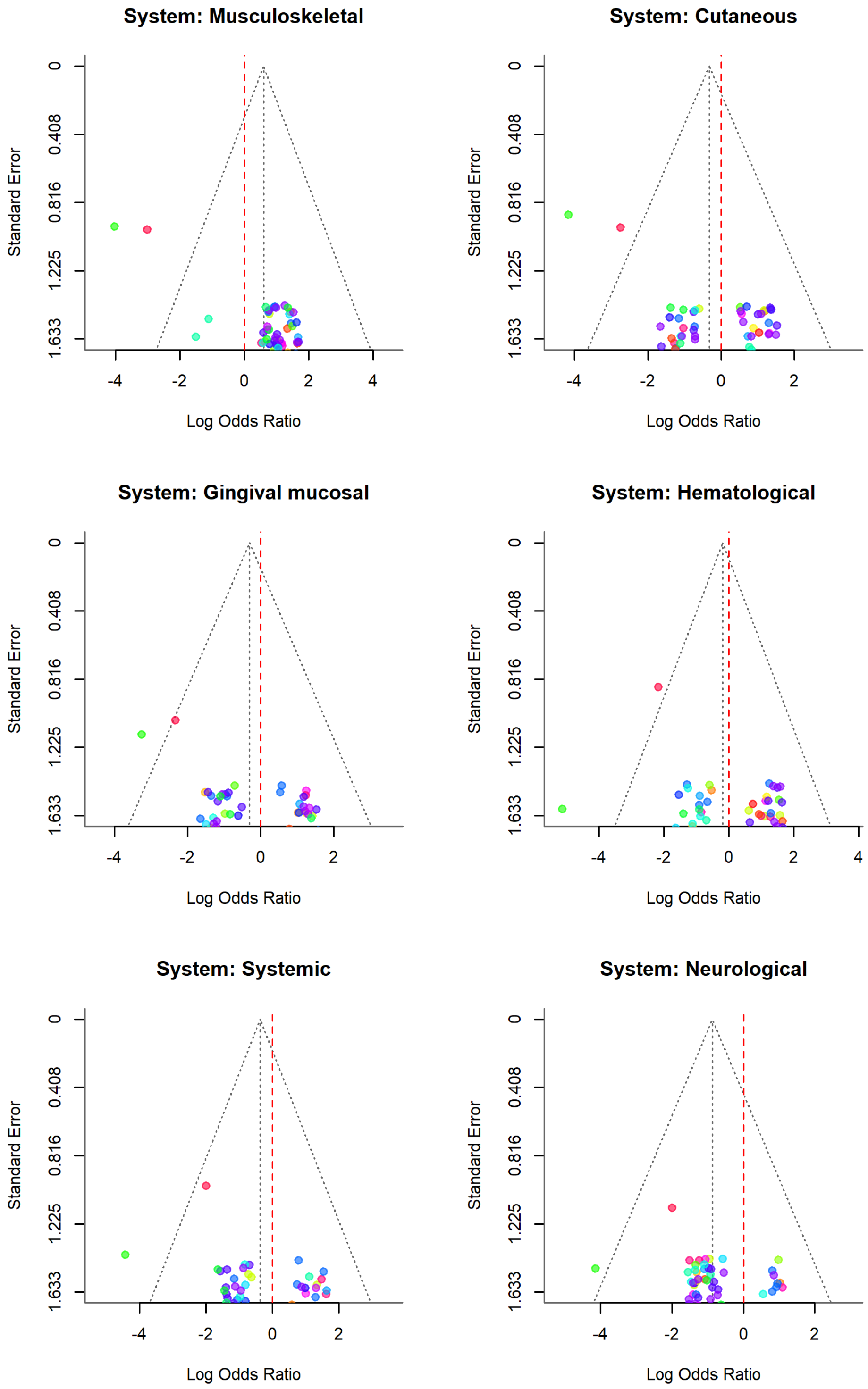

Supplement: Supplementary file 1 [file nutrients-18-02213-s001.zip › nutrients-4308267-supplementary.pdf]
